# Incidence and Characteristics of Pediatric Eosinophilic Esophagitis: A Midwestern State Analysis

**DOI:** 10.3390/children12020248

**Published:** 2025-02-19

**Authors:** Jose L. Zamora-Sifuentes, Andrew Rorie, Sharad Kunnath, Rosemary Pauley, Andrew Huang Pacheco, Russell Hopp

**Affiliations:** 1 Division of Allergy & Immunology, Department of Internal Medicine, University of Nebraska Medical Center, Omaha, NE 68198, USA; 2 Division of Gastroenterology, Department of Pediatrics, Boys Town National Research Hospital, Omaha, NE 68010, USA; 3 Division of Gastroenterology, Department of Pediatrics, Children’s Nebraska, Omaha, NE 68114, USA; 4 Division of Pulmonology, Allergy & Sleep Medicine, Department of Pediatrics, Children’s Nebraska, Omaha, NE 68114, USA

**Keywords:** Eosinophilic esophagitis, EoE, pediatrics, eosinophilic gastrointestinal diseases, EGID

## Abstract

Background: Eosinophilic esophagitis (EoE) is a chronic disease defined by esophageal dysfunction and >15 eosinophils per high-power-field on biopsy. Despite its increased incidence across the United States, studies evaluating its incidence at any state level are lacking. Methods: Record review of pediatric patients (<18 years) newly diagnosed with EoE based on ICD coding seen at the main two pediatric gastroenterology centers in the state: Children’s Nebraska (1 January 2016–31 December 2022) and Boys Town National Research Hospital (1 January 2022–31 December 2022). Data included demographics, age, and zip codes. Descriptive analysis focused on Nebraska residents. Results: The average point incidence of EoE between 2016 and 2022 was 10.84/100,000 inhabitants based on data from Children’s Nebraska. Considering both centers, the point incidence in Nebraska for 2022 was 32.45/100,000 inhabitants. Caucasians were 3.7 times more likely to be affected and older at time of diagnosis (average 9.7 years) compared to African Americans (7.0), Hispanics (7.4), and Asians (4.4). Conclusions: This is the first study evaluating the incidence of EoE in a specific U.S.A state. Studies at the state level are important to direct policy and interventions aiming limit its burden in communities.

## 1. Introduction

Eosinophilic esophagitis (EoE) was first described in the 1990s as a disorder of the esophagus characterized by severe esophageal symptoms, such as dysphagia in adults and refractory gastroesophageal reflux disease (GERD)-like symptoms in children, accompanied by dense eosinophilic infiltrates [1,2]. Since these early observations, EoE has now been defined as a chronic disease characterized by symptoms of esophageal dysfunction (e.g., dysphagia, odynophagia, failure to thrive) and an esophageal biopsy with at least 15 eosinophils per high-power field (HPF) after exclusion of non-EoE disorders that could contribute to esophageal eosinophilia [3]. It is the most common form of eosinophilic gastrointestinal diseases (EGIDs) with an overall prevalence of 34.2 cases per 100,000 inhabitants [4,5].

EoE predominantly affects male patients of Caucasian ancestry [6,7]. Multiple studies have also suggested that EoE more commonly affects adults [5,6]. Navarro and colleagues estimated that the overall pooled prevalence of EoE in adults was 42.2 cases per 100,000 inhabitants (95% CI, 31.1–55; I^2^ = 99.9%) [5]. In contrast, children aged < 16 years had an overall reported prevalence of 34.4 cases per 100,000 inhabitants (95% CI, 22.3–49.2; I^2^ = 99.8%) with an incidence rate of 6.6 cases per 100,000 inhabitants/year [5,6]. Despite the increased likelihood of developing EoE in adults, the incidence of EoE is increasing across all age groups [5]. Indeed, the incidence of EoE in children increased from 5.1 cases to 6.6 cases per 100,000 inhabitants/year between 2014 and 2018 [5,6].

Multiple studies have evaluated the incidence of EoE in the United States, with some of them going as far as evaluating its prevalence based on regional distribution (East, South, Midwest, and West) [8,9]. Interestingly, Dellon and colleagues found an increased prevalence of EoE in the Midwest compared to the remainder of the United States, with a combined prevalence of 50.7 cases per 100,000 for adults and children [9]. Nevertheless, studies at the state level are lacking and the overall prevalence and incidence of EoE in the state of Nebraska, especially amongst children, remains to be identified.

The aims of the present study are to identify the incidence of new cases of pediatric EoE in the state of Nebraska and its geographical distribution, as well as the demographic characteristics of the affected population. This data is of importance to identify the burden that EoE plays at a state level and to properly allocate resources to reduce its morbidity at a specific state level.

## 2. Materials and Methods

We performed a multicenter retrospective medical record review of pediatric patients (<18 years of age) newly diagnosed with eosinophilic esophagitis (EoE) in the 2 largest regional centers with pediatric gastroenterology in the state of Nebraska: Children’s Nebraska (Omaha, NE, USA) and Boys Town National Research Hospital (Omaha, NE, USA). A new diagnosis of EoE was defined as the first encounter in medical records, inpatient or outpatient, with ICD-10 code of eosinophilic esophagitis (K20.0). Data collected included age at time of diagnosis, sex, race, and zip code for patients under 18 years of age seen at Children’s Nebraska between 1 January 2016 and 31 December 2022, and at Boys Town National Research Hospital between 1 January 2022 and 31 December 2022. Data from 2022 obtained from Children’s Nebraska and Boys Town National Research Hospital was combined and analyzed to better represent the overall point incidence of EoE in the state of Nebraska and surrounding areas for that year.

A total of 449 patients were seen in Children’s Nebraska with an ICD-10 code of eosinophilic esophagitis between 1 January 2016 and 31 December 2022. Out of these 449 patients, only 367 of them were from Nebraska and the remaining 82 were excluded from analysis due to residing out-of-state. The patients seen in Children’s Nebraska and Boys Town National Research Hospital for the year 2022 were combined to give a more recent estimate of findings. A total of 189 patients were seen at both institutions during the year 2022. Due to residing out of state, 30 of these patients were excluded from the study, leaving a total of 159 patients for the year 2022.

Zip codes were used to determine the regional distribution (e.g., county, state) of EoE cases seen in both academic centers and plotted as a map chart. The overall rough point incidence, incidence by sex, and incidence by region were identified within the database. Data were analyzed using descriptive statistics to obtain population percentages for sex, race, and average age at time of diagnosis. The rough point incidence was calculated by using the total number of new EoE cases identified each year compared to the proportion of the population under the age of 18. This was subsequently expressed as a proportion to 100,000 inhabitants. Population estimates and percentages of population under 18 years of age were obtained from the U.S. Census Bureau. Given the limitations in calculating population estimates in specific age groups, and a relative stable population growth for the state of Nebraska, the percentage of patients under 18 years of age established in the 2020 US Census Bureau was used as a reference and across each year of the study. This study was approved by an Institutional Review Board at each facility.

## 3. Results

**Figure 1:**
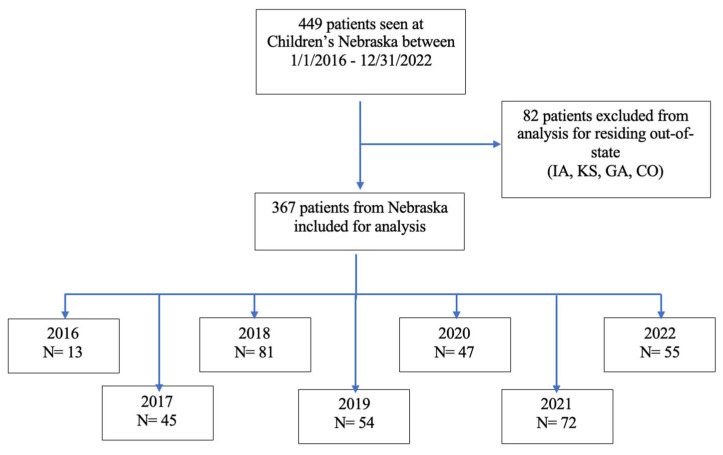
Summary of subjects seen at Children’s Nebraska included for single-center analysis.

A total of 449 patients were seen in Children’s Nebraska from 1 January 2016 to 31 December 2022 who met inclusion criteria, 336 males and 114 females (Figure 1).

**Figure 2:**
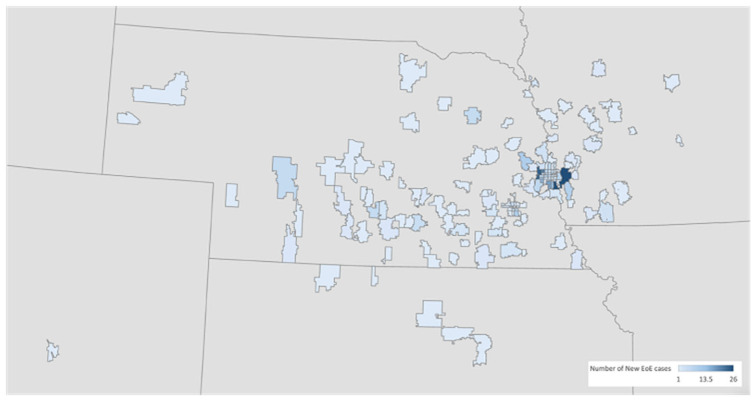
Regional heat map of new cases of eosinophilic esophagitis identified between 2016 and 2022 at Children’s Nebraska and in 2022 at Boys Town National Research Hospital. Plotting was done based on zip code of residence at the time of diagnosis. Georgia plotting not visualized.

Patients represented a total of 164 zip codes in five different states, Nebraska (NE), Iowa (IA), Kansas (KS), Georgia (GA), and Colorado (CO) (Figure 2).

For this study, only patients from Nebraska were considered for data analysis. This led to a total of 367 patients, 278 males and 89 females. The male-to-female ratio was 3.1. Individuals were categorized as Asian, Black or African American, Hispanic or Latino, White, or Multiracial. A total of 282 Caucasians were affected compared to 78 non-Caucasians, which included 28 patients self-identifying as Black or African American, 20 as Hispanic, 7 as Asian, 28 as Multiracial, and 1 as unknown/refused to provide information (Table 1).

**Figure 3:**
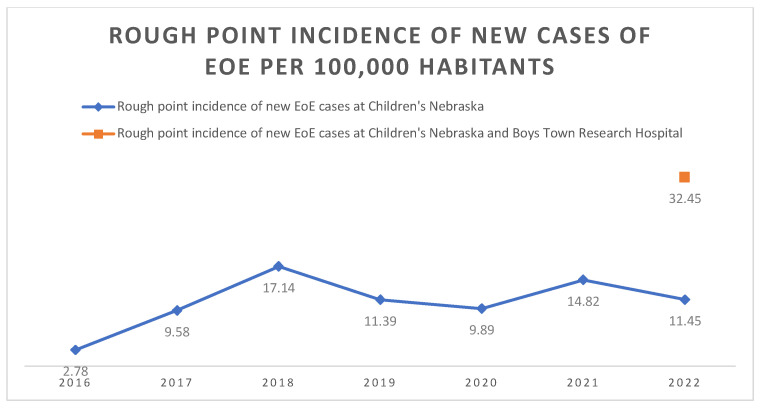
Rough point incidence of new EoE cases in Nebraska from a single academic center (Children’s Nebraska) and two academic centers (Children’s Nebraska and Boys Town National Research Hospital).

The average age at the time of diagnosis for all subjects was 9.2, with an age of 9.7 years for Caucasians, 7.0 for Blacks or African Americans, 7.4 for Hispanics, and 4.4 for Asians. Those identifying as multiracial had an average of 7.8 years at time of diagnosis. Caucasians were 3.7 times more likely to be affected than non-Caucasians. The average point incidence of EoE in the state of Nebraska based on EoE cases from patients living in the state and seen in Children’s Nebraska was 10.84 cases per 100,000 inhabitants from 2016 to 2022 (Figure 3).

A second, different set of 120 patients seen at Boys Town National Research Hospital from 1 January 2022 to 31 December 2022 was identified. Most patients were from Nebraska (104 patients), with the remainder residing in Iowa (16 patients). The patients from Nebraska were 76 males and 28 females, with a male-to-female ratio of 2.71. Most patients identified as Caucasian (89 patients), 6 as Black or African American, 2 as Hispanic or Latino, 4 as Asian, 2 as Multiracial, and 1 as unknown/refused to provide information. The average age at the time of diagnosis in Boys Town National Research Hospital was 10.79.

**Figure 4:**
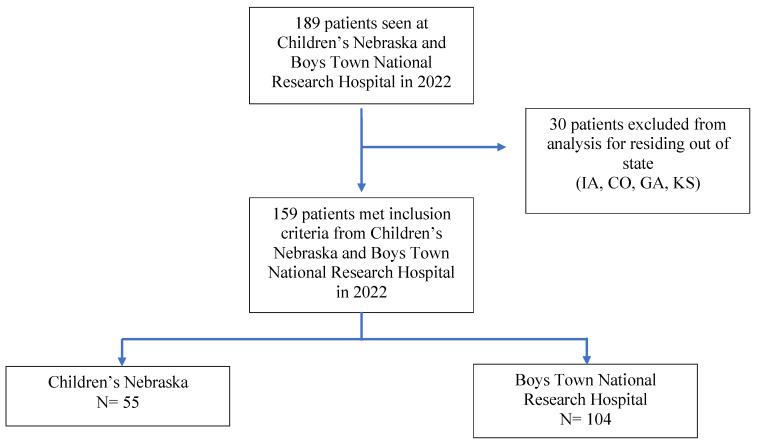
Summary of subjects seen at Children’s Nebraska and Boys Town National Research Hospital during the year 2022 and included for multi-center analysis.

The combined data for both centers for the year of 2022 showed a total of 159 patients with residency in Nebraska, 121 males and 38 females (Figure 4).

The male-to-female ratio was 3.18. The demographic breakdown included 132 patients self-identified as Caucasians, 9 as Black or African American, 3 as Hispanic, 6 as Asian, 8 as Multiracial, and 1 as unknown/refused to provide information (Table 2).

Caucasians were 4.9 times more likely to be diagnosed with EoE. The average age at the time of diagnosis was 10.1 years. Patients represented 74 different zip codes in NE, with most new cases in 2022 occurring in Douglas County, Lancaster County, and Sarpy County. Based on the data from both centers, the overall point incidence of EoE in the state of Nebraska was 32.45 cases per 100,000 inhabitants for the year 2022.

## 4. Discussion

Nebraska is a large, mostly rural state with a population of nearly 2 million. Despite its size, most of the population resides in urban and suburban areas in Eastern Nebraska, particularly those in Douglas County, Lancaster County, and Sarpy County [10]. Due to the nature of the state and its population distribution, almost all the specialty care for pediatric gastroenterology in the state is provided in the only two pediatric specialty centers in the state, Children’s Nebraska and Boys Town National Research Hospital. These two regional pediatric gastroenterology centers provide most of the pediatric eosinophilic esophagitis (EoE) care in Nebraska, providing a unique opportunity to determine incidence of EoE statewide. With the incidence of EoE increasing across the globe, understanding the incidence of EoE in a region is pivotal in allocation state resources to minimize the burden and morbidity of EoE in the community. To the best of our knowledge, this is the first epidemiological study evaluating the geographical distribution of EoE and its incidence amongst children in the state of Nebraska.

This study further supports the observation that EoE affects males more often than females at a ratio of approximately 3:1. Data from 2016 to 2022 also reinforced that Caucasians were more likely to be affected compared to other races and ethnicities by even a larger ratio. Interestingly, they were also diagnosed at a later age. While most minorities were diagnosed around age 7, Caucasians were diagnosed almost 2.5 years later, at an average age of 9.7 years. This contrasts the findings of Mahon and colleagues, who conducted a study in an inner-city, low-income pediatric outpatient center and noted that Black or African Americans were older at time of diagnosis, with a median age of 13 years [11]. The retrospective nature of this study limits the evaluation of additional factors that could explain this difference. However, it is possible that differences in socioeconomic status and environmental exposures amongst these groups played a role in the development of EoE in different timelines. The effect of environmental exposures is such, for example, that exposure to cats and dogs in the home during infancy has been inversely associated with EoE (adjusted Odds Ratio [aOR] = 0.48, 95% CI = 0.34–0.97) [12]. Nevertheless, the impact of environmental exposures in EoE is not only limited to environmental allergens. For example, Jensen et al. have shown an increased association between maternal and infant antibiotic and acid suppressant use with the development of EoE, further highlighting the complexity behind the pathogenesis of this disease [13].

This study showed that the highest number of new EoE cases in the state of Nebraska were observed in Douglas County, Sarpy County, and Lancaster County, whereas the largest number of new cases of EoE outside of Nebraska were found in Pottawattamie County, Iowa (Figure 2). These are also the counties with the largest population in a 125-miles radius from Omaha, the largest city in Nebraska.

The yearly rough incidence of pediatric EoE in the entire state of Nebraska was estimated by using the aggregate data for 2022 from the only two pediatric gastroenterology centers in the state. This was conducted in an effort to obtain the most recent available point incidence for the state. Compared to the yearly incidence reported in previous studies for the general pediatric population, the rough incidence in Nebraska was significantly elevated [5]. While the combined data from two academic centers was only reviewed for the year 2022, the yearly incidence of EoE based on data from Children’s Nebraska alone appears to have drastically increased since 2016. Indeed, the yearly point incidence for the state of Nebraska has remained above that of the pediatric average by nearly two-fold since 2018 based on data from Children’s Nebraska alone (Figure 4). Despite the significant increase in point incidence, particularly compared to that of 2016, the yearly incidence has remained grossly stable following 2018. The reason behind such a low incidence in 2016 is unclear as Children’s Nebraska has been an Eosinophilic Gastrointestinal Diseases (EGID) center since 1999. Furthermore, there were no significant changes in the approach towards EoE during this time that could further explain these numbers. On the other hand, it is possible that the drop in incidence seen in 2020 was due to the additional restrictions and precautions required to perform endoscopies during the COVID-19 pandemic, as well as parental concerns towards seeking medical care during the pandemic. This likely explains the increase point incidence seen for the year 2021, as those patients who avoided seeking medical care during the beginning of the pandemic were finally able to receive medical evaluation, with the numbers in 2022 returning close to pre-pandemic levels.

Additional studies further evaluating the prevalence of EoE per county population could prove useful for identifying populations at risk of developing EoE, as well as risk factors that could predispose children to a higher incidence. Recent studies have shown that, while EoE affects predominantly Caucasians and non-Hispanic patients in urban and higher socioeconomic status, Black children, and those in rural, underprivileged settings tend to show a higher burden of disease based on the need for feeding therapy and dilation at an earlier age [14]. This finding should be of extreme importance to the state of Nebraska given that the data pooled at Children’s from 2016 to 2022 suggests that Black or African American, Hispanic or Latino, Asian, and Multiracial children are diagnosed at an earlier age compared to their Caucasian counterparts.

This study has several limitations, particularly its retrospective and descriptive nature. Patients were identified under the premise that the ICD-10 code K20.0 was accurately used after first encountering the diagnosis of EoE. However, this is limited by the possibility of a patient having been previously diagnosed with EoE at an outside facility, which could lead to a possible year-to-year overlap. Nevertheless, the overall incidence trend in Nebraska is on an increase and the possibility of an overlap is limited by the reduced availability of pediatric subspecialty care in the state of Nebraska managing cases of EoE. In addition, this study does not factor in endoscopic nor pathology results into consideration and only utilizes ICD coding to identify cases of EoE. Additionally, other risk factors such as the presence of atopy or environmental exposures were not examined.

## 5. Conclusions

The incidence of eosinophilic esophagitis amongst children continues to rise. This study highlights that the point incidence of pediatric eosinophilic esophagitis in the state of Nebraska is higher than that of the general population. Furthermore, African American, Hispanic, and Asian patients were diagnosed with EoE at an earlier age despite EoE being more common amongst Caucasians. Given that new cases of EoE continue to rise in the United States, it is fundamental to understand its prevalence and burden in individual communities. Further epidemiological studies can help identify its regional distribution and risk factors favoring the development of EoE to allow for outreach and directed intervention, limiting morbidity in the pediatric population.

## Figures and Tables

**Table 1:** Demographics of patients from Nebraska seen in Children’s Nebraska with new diagnoses of eosinophilic esophagitis between 1 January 2016 to 31 December 2022.

Characteristics	Population Number (%)
**Sex**	
Male	278 (75.7)
Female	89 (24.3)
Total	367 (100)
**Race**	
Caucasian	289 (78.8)
Non-Caucasian	78 (21.2)
Black or African American	28
Hispanic	20
Asian	7
Multiracial	29
Unknown/Refused	1
**Average age at time of diagnosis**	9.2
Caucasian	9.7
Black or African American	7.0
Hispanic	7.4
Asian	4.4
Multiracial	7.8

**Table 2:** Demographics of patients from Nebraska seen in Children’s Nebraska and Boys Town National Research Hospital with a new diagnosis of eosinophilic esophagitis seen between 1 January 2022 and 31 December 2022.

Characteristics	Population Number (%)
**Sex**	
Male	121 (76.1)
Female	38 (23.9)
Total	159
**Race**	
Caucasian	132 (83.0)
Non-Caucasian	27 (17.0)
Black or African American	9
Hispanic	3
Asian	6
Multiracial	8
Unknown/Refused	1
**Average age at time of diagnosis**	10.1

## Data Availability

The original contributions presented in this study are included in the article. Further inquiries can be directed to the corresponding author.

## Abbreviations

EoE: eosinophilic esophagitis; GERD, gastroesophageal reflux disease; EGIDs, eosinophilic gastrointestinal diseases; HPF, high-power field.
